# Biochemical efficacy, molecular docking and inhibitory effect of 2, 3-dimethylmaleic anhydride on insect acetylcholinesterase

**DOI:** 10.1038/s41598-017-12932-0

**Published:** 2017-10-02

**Authors:** Kabrambam D. Singh, Rajendra K. Labala, Thiyam B. Devi, Ningthoujam I. Singh, Heisnam D. Chanu, Sonia Sougrakpam, Bunindro S. Nameirakpam, Dinabandhu Sahoo, Yallappa Rajashekar

**Affiliations:** 1 Insect Resources Laboratory, Animal Resources Programme, Institute of Bioresources and Sustainable Development, Department of Biotechnology, Govt. of India, Takyelpat, Imphal-795001, Manipur, India; 2 Distributed Information Sub-Centre, Institute of Bioresources and Sustainable Development, Department of Biotechnology, Govt. of India, Takyelpat, Imphal-795001, Manipur, India; 3 Microbial Resources Programme, Institute of Bioresources and Sustainable Development, Department of Biotechnology, Govt. of India, Takyelpat, Imphal-795001, Manipur, India

## Abstract

Evolution of resistance among insects to action of pesticides has led to the discovery of several insecticides (neonicotinoids and organophosphates) with new targets in insect nervous system. Present study evaluates the mode of inhibition of acetylchlonesterase (AChE), biochemical efficacy, and molecular docking of 2,3-dimethylmaleic anhydride, against *Periplaneta americana* and *Sitophilus oryzae*. The knockdown activity of 2,3-dimethylmaleic anhydride was associated with *in vivo* inhibition of AChE. At KD_99_ dosage, the 2,3-dimethylmaleic anhydride showed more than 90% inhibition of AChE activity in test insects. A significant impairment in antioxidant system was observed, characterized by alteration in superoxide dismutase and catalase activities along with increase in reduced glutathione levels. Computational docking programs provided insights in to the possible interaction between 2,3-dimethylmaleic anhydride and AChE of *P*. *americana*. Our study reveals that 2,3-dimethylmaeic anhydride elicits toxicity in *S*. *oryzae* and *P*. *americana* primarily by AChE inhibition along with oxidative stress.

## Introduction

Major insecticides developed in the last 60 years act on one of the following targets; acetylcholinesterase, an enzyme of critical importance in the transmission of nerve impulse (organophosphorus and carbamates), voltage-gated sodium channels across the nerve membrane (pyrethroids and DDT), and the acetylcholine receptor (neonicotinoids)^1–5^. Acetylcholine (ACh) is a one of major neurotransmitter involved in neurotransmission^6^. ACh is produced from acetylating reaction with choline and Acetyl-CoA by the enzyme choline acetyltransferase. In the synaptic cleft, ACh is degraded by an enzyme acetylcholinesterase forming acetate and choline, which are recaptured by the pre-synaptic neurons^7^. Organophosphates and carbamates insecticides are potent inhibitors of the enzyme acetylcholinesterase.

Insect pest management is facing the economic and ecological problems worldwide due to the human health, environmental hazards and pest species developing insecticides resistance caused by extensive use of chemical insecticides^8–10^. Discovery of novel effective insecticidal compounds is needed to combat the increasing resistance rates. Botanicals contain active insecticidal phytochemicals, which are considered as alternatives to hazardous and non biodegradable chemical insecticides^11–13^. Essential oils and plant volatile organic compounds are good sources for developing insect pest control agents because they are known to have many bioactivities including insecticidal, repellents, antifeedants, ovicidal and insect growth regulators activity against insect pests^14–17^. The main advantage of plant volatile organic compounds is its highly volatile nature. High volatility reduces the residue problems and, easily degradable, eco-friendly and less toxic in mammals^18^ and used in the indigenous traditional medicines in most of tropical areas^19^.

The evaluations of insecticidal efficacy of plant derived products are based on use in the traditional practices in pest management, which forms a basis for an ideal approach in the development of newer insecticides from plants. One of the plants is *Colocasia esculenta* var esculenta (L.) Schott, commonly known called as Taro, geographically occurs throughout India and it is cultivated worldwide. It is an annual herbaceous plant with a long history of usages in traditional treated medicine such as asthma, arthritis, diarrhoea, neurological and skin disorders and further, juice of corm is widely used for treatment of bodyache and baldness^20^. In North East India, people have practise to consume corm and root stock of *C*. *esculenta* as food from many centuries and there were no reports on adverse effect on health.

Recently, Rajashekar *et al*.^21^ isolated 2,3-dimethylmaleic anhydride, a novel biofumigant molecule from root stock of *C*. *esculenta* (L.) Schott, which is highly toxic to various stored grain insects and house fly by fumigation^21^. Further, this molecule has no adverse effect on seed germination which makes it highly desirable for grain/seed protection against stored grain insect pests. The insect toxicity of 2,-3-dimethylmaleic anhydride in the fumigant bioassay was analyzed based on the symptoms and behaviours and the results indicated its neurotoxic nature similar to those produced by organophosphates. In this paper, we investigate the toxicity and the mode of inhibition of acetylcholinesterase by 2,3-dimethylmaleic anhydride. We also measured the effect of antioxidant defense system (SOD, GSH, CAT), involved in the toxicity aspect of its action in *Sitophilus oryzae* (L.) and *Periplaneta americana*, which leads us to postulate that these natural components represent the discovery of mode of action and oxidative imbalance. Subsequently, a molecular docking study was investigated to enlighten the possible molecular interactions between 2,3-dimethylmaleic anhydride and the enzyme acetylcholinesterase (AChE) of *P*. *americana*.

## Results

### *In vivo* inhibition of acetylcholinesterase in relation to insect toxicity

#### Dose-response

**Figure 1:**
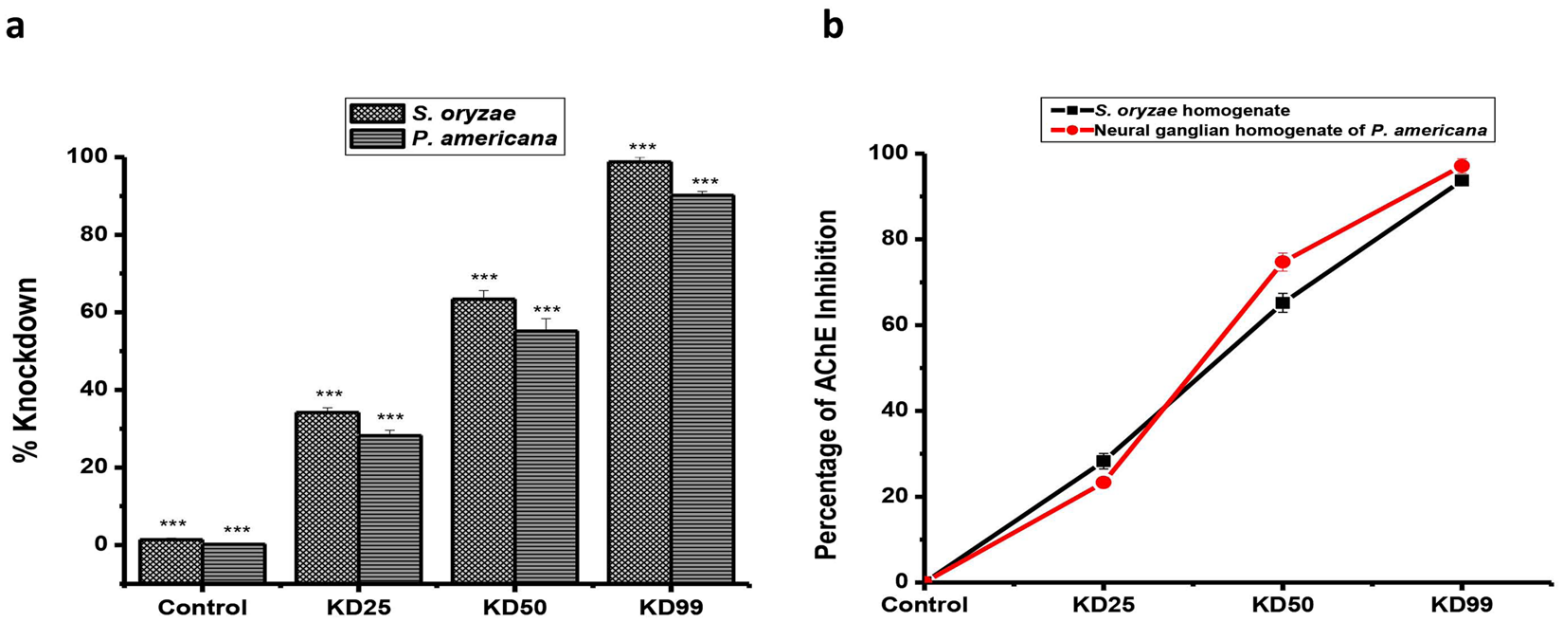
Dose-dependent *in vivo* inhibition of acetlycholinesterase by 2,3-dimethylmaleic anhydride in relation to insecticidal activity in the American cockroach (*P*. *americana*) and rice weevil (*S*. *oryzae*) (n = 4, error bars, s.e.m.). (**a**) % Knockdown effect. (**b**) % of AChE inhibition.

Activity of acetylcholinesterase in insects exposed to KD_25_, KD_50_ and KD_99_ doses of 2,-3-dimethylmaleic anhydride, was markedly suppressed in a dose-dependent in neural ganglion of American cockroach and homogenate of rice weevil (Fig. 1a,b). The *in vivo* enzyme inhibition was dose-dependent and correlated with the knockdown effect measured at 2 h of exposure in the fumigation bioassay.

#### Time-dependent study

In *S*. *oryzae* (3 µg/L) and *P*. *americana* (10 µg/L) treated with a single KD_50_ dose of 2,-3-dimethylmaleic anhydride at various exposure times (0–120 min), inhibition of acetylcholinesterase increased with time and correlated with the knock down effect (Tables 1 & 2).

**Table 1:** The knockdown activity of KD_50_ dose of 2,3-dimethylmaleic anhydride against *P*. *americana and S*. *oryzae* at different exposure period.

Exposure period (min)	% knockdown (mean ± SE)			
	*P*. *americana*		*S*. *oryzae*	
	Control	Treated	Control	Treated
30	0	12.3 ± 2.4^a^	0.8 ± 0.2^a^	27.9 ± 2.4^a^
60	0	48.5 ± 1.4^b^	2.2 ± 0.4^b^	52.3 ± 1.4^b^
90	1.2 ± 0.1^a^	80.3 ± 1.2^c^	3.7 ± 0.4^c^	78.3 ± 1.2^c^
120	1.5 ± 0.2^b^	90.2 ± 2.8^d^	5.2 ± 0.8^d^	92.3 ± 1.4^d^
150	1.8 ± 0.1^c^	98.2 ± 1.2^e^	6.5 ± 0.4^e^	96.9 ± 2.2^e^

The values were expressed within a column indicate that mean ± standard error of four replicates and significantly different (P < 0.05) by Newman-Keuls test.

**Table 2:** *In vivo* inhibition of KD_50_ of 2,3-dimethylmaleic anhydride on acetlycholinesterase in neural ganglion of *P*. *americana* and *S*. *oryzae* homogenate at different exposure.

Exposure period (min)	% of AChE inhibition (mean ± SE)			
	*P*. *americana*		*S*. *oryzae*	
	Control	Treated	Control	Treated
30	2.1 ± 0.4^a^	40.5 ± 2.4^a^	2.4 ± 0.2^a^	47. 2 ± 2.4^a^
60	4.2 ± 0.2^b^	80.3 ± 2.2^b^	6.3 ± 0.4^b^	63.2 ± 1.8^b^
90	5.8 ± 0.5^c^	90.2 ± 3.4^c^	7.2 ± 0.4^c^	87.4 ± 1.2^c^
120	8.3 ± 0.4^d^	98 ± 2.2^d^	8.8 ± 0.6^d^	90. 1 ± 3.2^cd^
150	10.2 ± 1.2^e^	98.2 ± 2.4^de^	13.6 ± 2.4^e^	96.8 ± 1.2^e^

The values were expressed within a column indicate that mean ± standard error of four replicates and significantly different (P < 0.05) by Newman-Keuls test.

### *In vitro* inhibition of acetylcholinesterase

**Figure 2:**
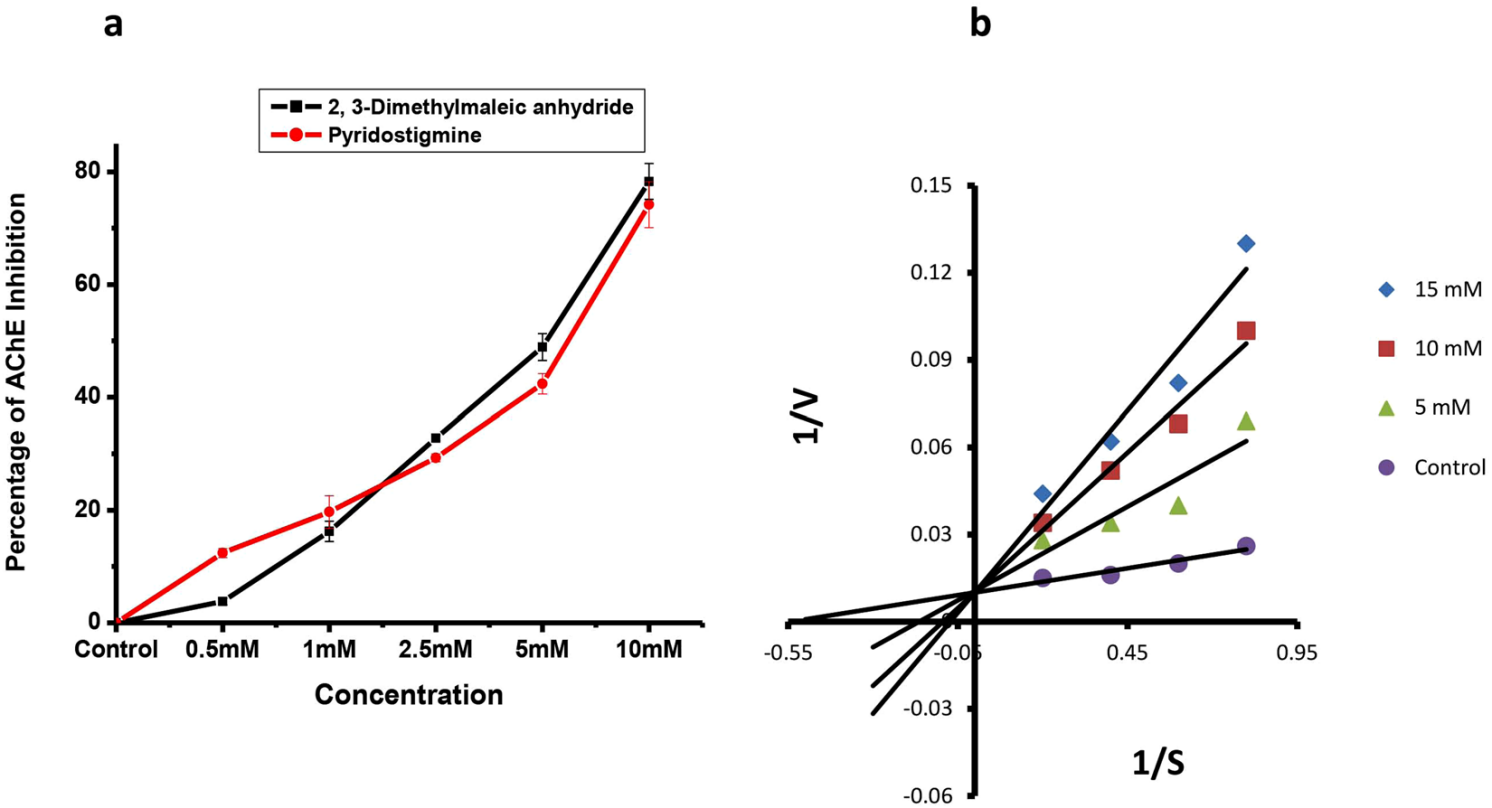
In *vitro* inhibition of acetlycholinesterase by 2,3-dimethylmaleic anhydride. (**a)** Inhibitory effect of acetlycholinestearse by 2,3-dimethylmaleic anhydride in comparison with that of pyridostigmine bromide in crude neural ganglion homogenate of *P*. *americana* (n = 4, error bars, s.e.m.). (**b**) Kinetics of *in vitro* inhibition of acetlycholinesterase in crude neural ganglion homogenate of *P*. *americana* (n = 4, error bars, s.e.m.). The double reciprocal (Linweaver-Burk) plot.

The *in vitro* inhibition of acetylcholinesterase was increased with the concentration of 2,3-dimethylmaleic anhydride in the cockroach (neural ganglion) crude homogenate and the inhibition efficiency of AChE from isolated compound is comparable to that of standard AChE inhibitor pyridostigmine bromide (Fig. 2a). In fact, the molecule exhibited dose-dependent AChE inhibitory effect with an IC_50_ value of 5.79 mM. Further the reciprocal plot (Fig. 2b) showed an increasing concentration of the substrate decreased the enzyme inhibition due to 2,3-dimethylmaleic anhydride. Kinetic studies showed that the K_m_ shifted with the increased concentration of the inhibitors indicating that the inhibition was competitive as evident from the Lineweaver-Burk plot (Fig. 2b).

### Effect of antioxidant enzyme system

**Figure 3:**
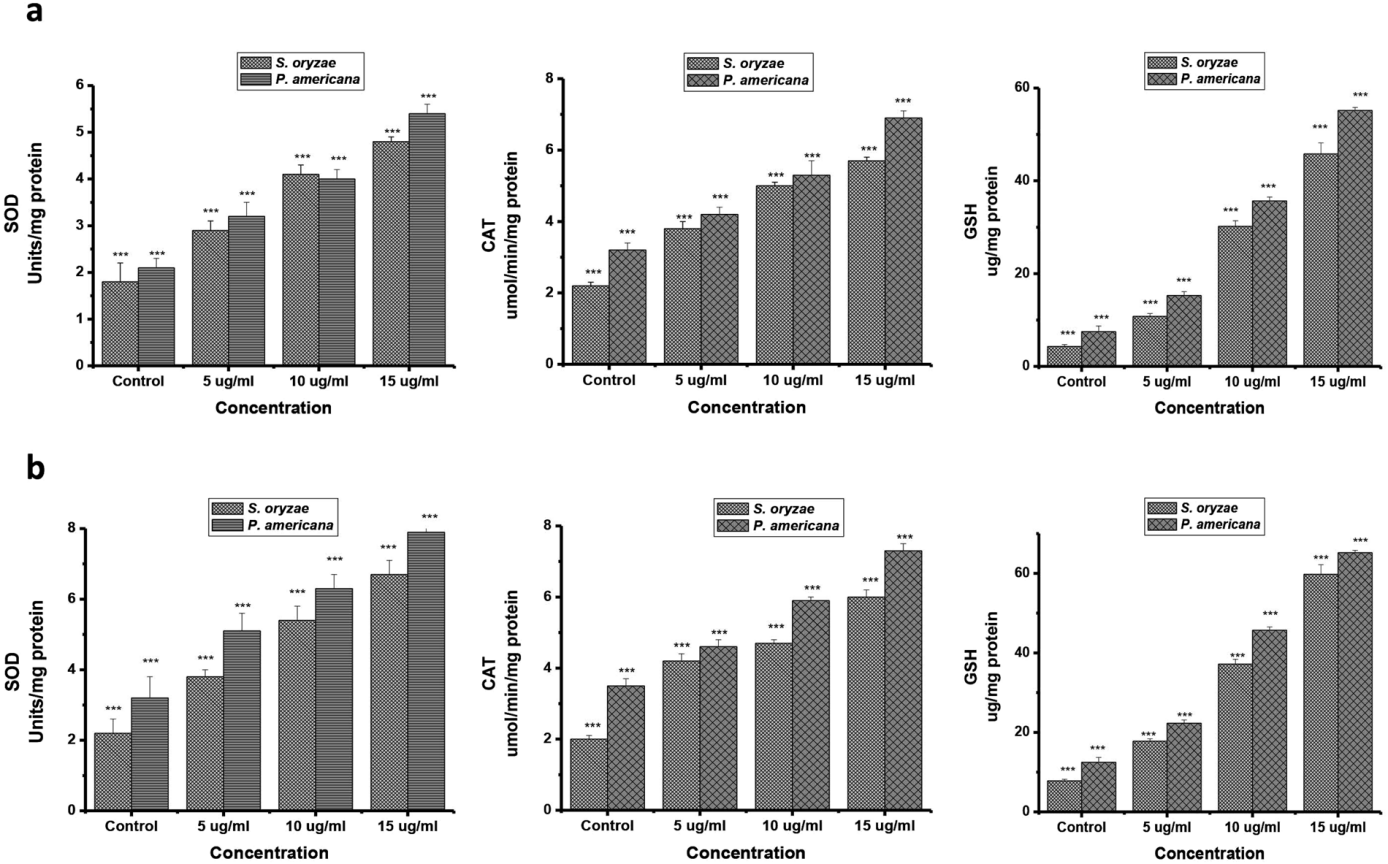
Effect of different concentrations of 2,3-dimethylmaleic anhydride on SOD, CAT activity and GSH content in *S*. *oryzae* and *P*. *americana* at (**a**) 12 h and (**b**) 24 h exposure (n = 4, error bars, s.e.m.).

The effect of 2,3-dimethylmaleic anhydride on the antioxidant enzyme system was marked and resulted in distinct variations in test insects. The SOD activity was significantly increased with the dose of 2,3-dmethylmaleic anhydride and in a time-dependent manner. SOD activity was increased significantly with increase in the dose of 2,3-dimethylmaleic anhydride and exposure period. At 15 µg/ml, SOD activity was significantly elevated about 290.2% with control in *S*. *oryzae*, following 24 h exposure, where as in case of *P*. *americana* a significant increase about 251.3% compared with control was observed following for 24 h exposure (Fig. 3a,b) respectively.

Similar trend was observed for CAT activity exposed to 2,3-dimethylmaleic anhydride. CAT activity was significantly elevated in treated insect with increasing concentration of the compound. At 15 µg/ml, CAT activity was predominately increased (290% compared with the control). While for *P*. *americana* a drastically increased about 229% compared with the control was observed following 24 h exposure (Fig. 3a,b).

Further, we observed parallel trend for GSH activity exposed to 2,3-dimethylmaleic anhydride. GSH activity was significantly increased in both treated insects (347% and 720.2 for *S*. *oryzae*, while in *P*. *americana* 360% and 475.3% respectively) compared with control was observed following 12 and 24 h exposure (Fig. 3a,b).

### Comparative homology modeling

**Figure 4:**
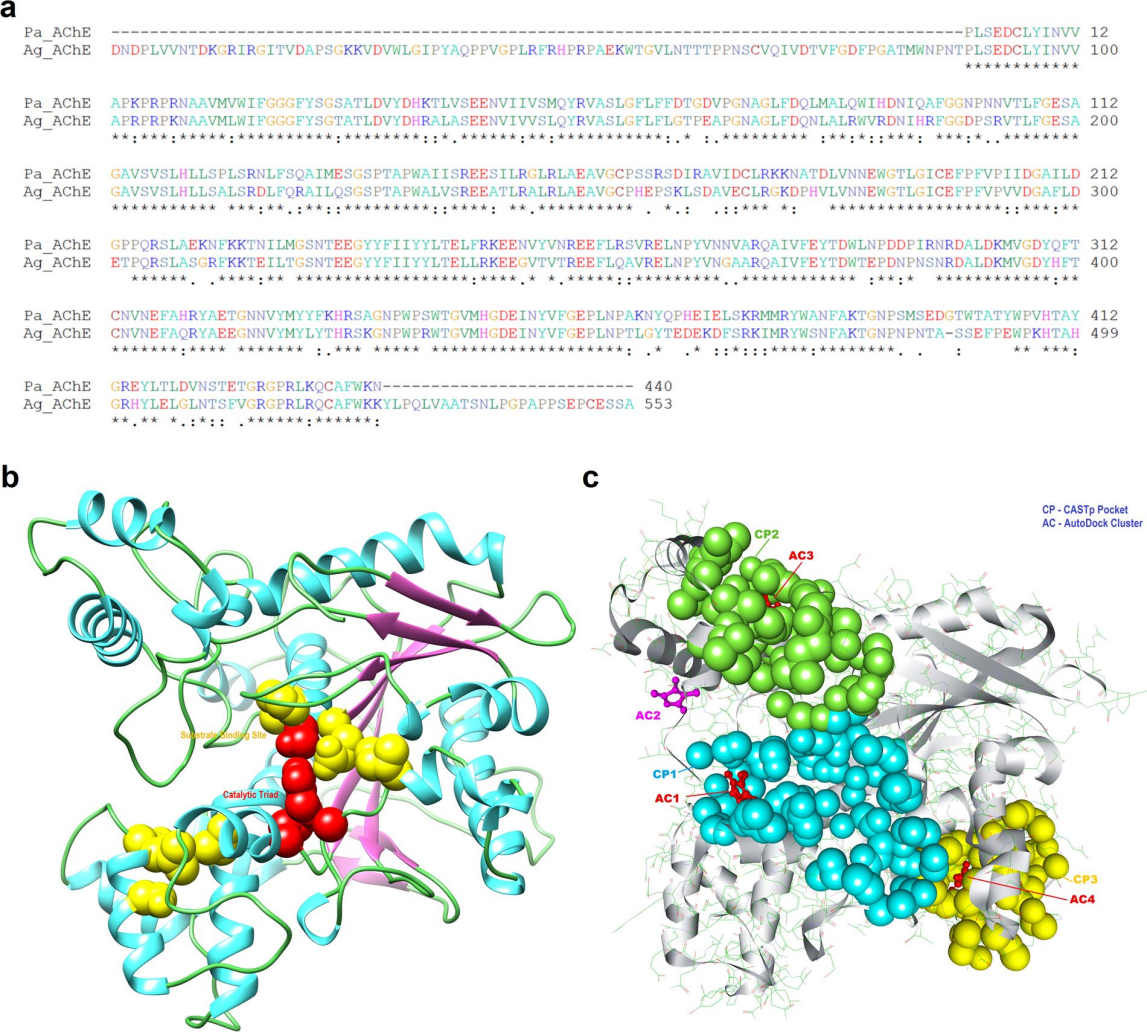
Target-template alignment and modelled structure of AChE with potential binding pockets: (**a**) *Pa_AChE*: AChE protein sequence of *P*. *americana* (ALJ10969.1) and *Ag_AChE*: AChE protein sequence of *Anopheles gambiae* (PDB ID: 5 × 61, Chain B). Amino acid positions are numbered on the right, conserved regions are indicated by (*), conserved substitutions are indicated by (:), semi-conserved substitutions are indicated by (.), and deletions by (−). (**b**) 3D homology model rendered by the SWISS-MODEL program presented in rounded ribbon (Helix-cyan, Strand-pink, Coil-light green). Substrate binding sites (yellow) and catalytic triad (red) are represented in spheres. (**c**) Major three CASTp identified pockets CP1 (cyan), CP2 (lime), and CP3 (yellow) represented in sphere. Top four AutoDock conformation clusters (ball & stick representation) AC1, AC3, and AC4 in color red, coinciding with CASTp pockets. AutoDock cluster AC2 in color pink, a separate binding site not overlapping with any CASTp pockets.

AChE Catalytic Subunit of *Anopheles gambiae* (PDB ID: 5 × 61, Chain B) at 3.4 Å resolution was chosen as the best available template to build the 3D structure for AChE with 99% query coverage and 73% similarity. Target-template alignment and its superimposed structure with the template 5HQ3 chain B are shown in Fig. 4a and Supplementary Fig. 1 respectively, while the generated 3D model with its substrate binding site and catalytic triad was shown in Fig. 4b. The GMQE score generated by the target-template alignment of the modelled protein is articulated between zero and one, and higher scores indicate increased structural reliability 48. The GMQE score for the modelled AChE was found to be 0.90, which indicates good model accuracy. Verification of the results, using different tools, consistently indicated a good quality of the predicted model. Stereo chemical quality of dihedral angles Φ against Ψ of amino acid residues in the predicted model was explored using Ramachandran plot (Supplementary Fig. 2a). The plot revealed 89.0% residues are within the most favoured regions (red), 10.0% of residues are within the additional allowed regions (yellow), 0.8% residues are within the generously allowed regions (beige), and only 0.3% residues of modelled protein are within the disallowed regions (white). Protein quality predictor (ProQ server) indicated LGscore of “extremely good model” and MaxSub of “very good model” quality measures. Z-score for the predicted model is within the range of scores typically found for the native proteins of similar size (Supplementary Fig. 2b) while the plot of residue energies revealed that entire calculated value was negative, where positive values correspond to problematic or erroneous parts of the input structure (Supplementary Fig. 2c). All of these results showed that the generated model is reliable and can be used for docking studies with higher confidence.

### Molecular docking analysis

**Figure 5:**
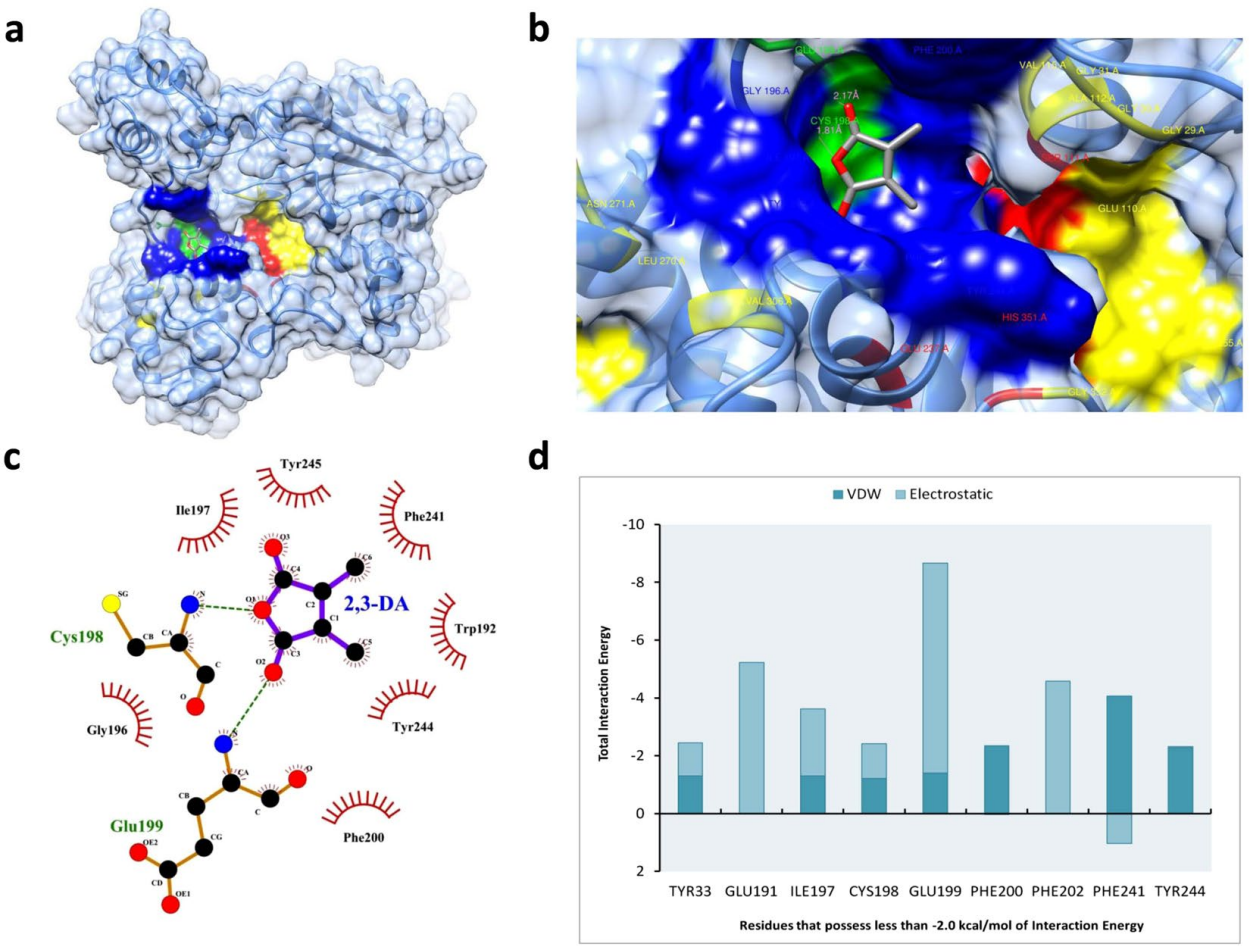
Summary of molecular docking analysis: (**a)** AChE and 2,3-dimethylmaleic anhydride docked complex, with solid surfaces representing hydrogen bonding sites (green), hydrophobic interactions (blue), substrate binding site (yellow), and catalytic triad (red). (**b)** 3D surface representation of 2,3-dimethylmaleic anhydride and AChE interactions with hydrogen bonding sites (green), hydrophobic interactions (blue), substrate binding site (yellow), and catalytic triad (red). (**c)** Schematic representation of the interaction in detail. (**d)** Plot showing total interaction energy (IE) possess by residues less than −2.0 kcal/mol of IE. (VDW: van der Waals interaction).

CASTp analysis of the predicted model of AChE identified three major pockets, named here as CP1 (CASTp Pocket 1), CP2, and CP3. (Fig. 4c). The details of the area covered and list of amino acids involved for each pocket is shown in Supplementary Table 1. We note that most of the residues involved in forming the substrate binding site (GLY29, GLY30, GLY31, GLU110, SER111, ALA112, VAL115, SER266, LEU270, ASN271, VAL306, GLY352, and ILE355) and catalytic triad (SER111, GLU237, and HIS351) of the modelled AChE are located in pocket CP1. Two sets of docking analysis were executed using the AutoDock 4.2 program; first, by involving the whole protein and second, only involving the focused area of the protein. First set of docking simulation allowed us to find and confirm potential binding sites for the ligand. The resulted multiple conformations of the AChE and 2,3-dimethylmaleic anhydride complexes were subjected to cluster analysis. Top four populated clusters from the analysis; named here as AC1 (AutoDock Cluster 1), AC2, AC3 and AC4 were taken for further investigations. It has been observed that, out of these four clusters, three clusters AC1, AC3, and AC4 confirmed the three major CASTp pockets CP1, CP2, and CP3 respectively as potential ligand binding sites. Besides, the remaining cluster AC2 did not overlap with any of the other sites hence identified as fourth potential binding site (Fig. 4c; Supplementary Table 1). AC1 cluster is formed near the designated substrate binding site inside CP1. Therefore, the potential binding site for further investigations was confirmed collectively by taking AC1 and CP1 sites into consideration. Further a new set of focused docking simulation was performed on this confirmed binding site. The resulted docking conformation from the second set of docking experiment having lowest binding energy of −5.25 kcal/mol is selected as the representative structure for the complex (Fig. 5a). There are total 153 atom contacts found between the protein and ligand. As shown in Fig. 5b,c 2,3-dimethylmaleic anhydride interacted with 9 amino acids forming hydrogen bonds with CYS198 and GLU199 with distances of 1.81 Å and 2.17 Å respectively. Further, the interaction energies (IE) between the protein and ligand were analysed for individual amino acids as well as overall. IE between the protein and ligand was equally shared by the electrostatic (IE: −18.50 kcal/mol) and van der Waals (IE: −19.16 kcal/mol) interactions. Residues that possess less than −2.0 kcal/mol of interaction energy were established (Fig. 5d). Out of them GLU199, GLU191, and PHE202 were found as contributor of polar or electrostatic interaction, whereas the PHE241, PHE200, and TYR244 were involved in van der Waals interactions.

## Discussion

Natural products could be an excellent source of novel pest control agents. An important attribute of natural product- based pesticide compounds is that they are relatively eco-friendly^21^^,^^22^. Among the many important insecticide classes, their origin is traceable to a natural product as in the case of pyrethoids, avermectins, decaleside; spinosads and the neonicotinoids^23–25^. Despite the diversity of natural chemistries, compounds with new modes of action have eluded discovery^11^^,^^26^. Although, insect control agents acting on targets other than nervous system such as insect growth regulators (e.g.: azadirachtin, JH analogues, ecodysone antagonists), have been developed, due to their lack of fumigant toxicity, they are not highly successful, but find a place in the integrated pest management scheme^27,28^.

2,3-dimethylmaleic anhydride the natural insecticides isolated and characterized by us from the root stock of *Colocasia esculenta*, were found to be toxic to a variety of insect species by the method of fumigation^21^. Our earlier studies showed that the insect toxicity of 2,3-dimethylmaleic anhydride was comparable to that of chemical insecticides (fumigants) and more potent than that of reported natural fumigants as evident by the LC_50_ values in fumigant bioassay^21^. Although, several natural compounds have been reported to exhibit fumigant toxicity, there is no comparative study of the toxicity of a natural compound with that of synthetic insecticides on insects in a fumigant bioassay. The nature of insect toxicity of 2,3-dimethylmaleic anhydride in the fumigant bioassay, based on the symptoms, indicated hyperactivity as seen in the increased movements followed by knockdown with legs showing over exciting movements indicating a neuro-muscular effect. The neurotoxic symptoms were similar to that of organophosphates.

A complete understanding or elucidation of the mode of action is of practical importance because it may provide newer opportunities for development of eco-friendly insecticides. Therefore, in the present study effect of 2,3-dimethylmaleic anhydride on AChE and antioxidant enzyme system was studied. Results clearly showed that AChE activity is drastically inhibited in insects exposed to 2,3-dimethylmaleic anhydride in fumigation bioassay. The *in vivo* inhibition closely correlates with insect toxicity of compound on increasing the concentration and exposure period. In addition, the in vitro studies reveal that, the molecule 2,3-dimethylmaleic anhydride showed potent AChE inhibitor and inhibition was competitive. AChE is an enzyme which breakdown the neurotransmitter acetylcholine to acetate and choline at the neuromuscular junctions^26^^,^^29^. Therefore, the inhibition of AChE activity leads to persistence of acetylcholine at the synaptic cleft (the space between two nerve cells) and cholinergic stress^30^, which could be one of the possible reasons of fumigant toxicity of 2,3-dimethylmaleic anhydride. Another probable reason is behavioral systems similar to that of insect exposed to carbamates. These two similar supportive observations have been reported for the most commercial available conventional insecticides such as organophosphates and carbamates^31^.

Further, it has been reported that oxidative stress is one of the possible mechanisms of the non target of commercial available pyrethriods, organochlorine and organophophates to the mammalian system^26,31,32^. Therefore, in the present investigation the effect of 2,3-dimethylmaleic anhydride on antioxidant enzyme system of insects were studied with dose-response and time-course dependent. The present result reveals that 2,3-dimethylmaleic anhydride causes significant impairment/imbalance in the antioxidant enzyme system (SOD and CAT) of both insects. In an organism, the SOD-CAT antioxidant system provides the first defense against oxygen toxicity. A delicate balance exists between the rate of formation of H_2_O_2_ via dismutation of superoxide by SOD activity and the rate of removal of H_2_O_2_ by catalase. Any impairment in this pathway, will affect the activities of other enzymes in the cascade^33,34^. Induction of antioxidant enzyme activities enhances metabolic detoxification and antioxidant defenses of the tissue, while their reduction results in oxidative stress^34,35^. In our study, 2,3-dimethylmaleic anhydride treated to insect led to reduced antioxidant capacity of the neural ganglion as evident from the decreased activity of all the antioxidant enzymes. However, John *et al*.^36^ and Aslanturk *et al*.^37^ reported that, variations on antioxidant enzyme system of treated insect due to the difference in biochemical and physiological response of treated insects. Further a significant increase in GSH level was recorded for the *S*. *oryzae* exposed to 2,3-Dimethylmaleic anhydride. GSH also serves as a substrate for glutathione peroxidises (GPx) and GST, primarily involved in the detoxification of toxic electrophilic metabolites via formation of GSH-conjugate^38^. During the enzymatic reaction catalyzed by GPx, GSH is oxidized to GSSG which is reduced back to GSH by GR. GR, therefore, plays a central role in maintaining the cellular GSH level. GSH plays an important role in protecting cells against reactive oxygen species (ROS) mediated injury by detoxification of lipid hydro peroxides formed due to oxidative damage^39^. Our results show drastic reduction in the activity of GSU in both test insects by 2,3-dimethylmaleic anhydride which could compromise the biochemical antioxidant defenses of the brain. Marked depletion of GSH and its dependent enzymes in the test insect by 2,3-dimethylmaleic anhydride indicates a major deleterious effect on the neural ganglian of insect. The results lead us to conclude that the oxidative imbalance may be involved in the toxic effects of 2,3-dimethylmaleic anhydride. Our results are the first report of a natural insecticide (biofumigant) might be associated inhibition of AChE activity and oxidative imbalance. The molecular mechanisms involved in the associated inhibition of AChE activity and oxidative imbalance that leads to the knockdown effect, the toxic outcome finally leading to mortality, are not clear at present. Further, our results imply that 2,3-dimethylmaleic anhydride may be associated inhibition of AChE activity and oxidative imbalance. However, further downstream events leading to the AChE inhibition need to be understood.

In addition, molecular docking may be reducing the understanding of the factors determining biological activity. Hence, we have investigated the possible binding modes of the 2,3-dimethylmaleic anhydride with AChE of *P*. *americana*. Since AutoDock program^40^ has been successfully applied for publishing docking studies with AChE^41^, it is utilized for our binding study. Absence of crystal structure of AChE protein of *P*. *americana* compelled us to perform the homology modelling to predict its 3D structure using SWISS-MODEL automated server. Best model generated from the server was further validated as described in results section. Although the ultimate availability of crystal structures of AChE protein of *P*. *americana* would improve the results of docking, the homology model predicted here acted as the preliminary step for testing 2,3-dimethylmaleic anhydride binding. CASTp finding of surface accessible pockets plus internal remote openings in the predicted model suggest that there are three major binding pockets covering very large volume (2982.2 Å^3^), with the largest pocket CP1 (1140.5 Å^3^) having most of the substrate binding sites and catalytic triad residues. By means of whole protein docking simulation we conclude that out of four major clusters formed, AC1 cluster of ligand conformations coincides with the pocket CP1 sites having most of the substrate binding site residues and catalytic triad. In this study, the intriguing finding is the different binding sites for 2,3-dimethylmaleic anhydride at AChE of *P*. *americana*. Focused docking on the CP1 binding site obtained a refined IE for the ligand. Evaluation of binding site residues involved in pocket configuration and those involved in hydrogen bond formation with 2,3-dimethylmaleic anhydride indicated that the ligand binds near the substrate binding site. By analysing the docking solutions, binding free energy and IE, we suggest that the 2,3-dimethylmaleic anhydride can attach to the substrate binding site of AChE through both electrostatic and van der Waals interaction as well as hydrogen bond, where the GLU199 played as main facilitator. As our experimental and computational findings corroborate each other, we can articulate that 2,3-dimethylmaleic anhydride is a potent inhibitor of AChE in *P*. *americana* for its activity. Nevertheless, this study reports the first binding analysis of 2,3-dimethylmaleic anhydride with AChE of *P*. *americana*.

The findings of present investigation reveal that, the, 2,3-dimethylmaleic anhydride is potent AChE inhibitor extracted from *C*. *esuclenta*. The knock down activity 2,3-dimethylmaleic anhydride is concomitant in vivo inhibition of AChE in both test insects are dose-response and time dependent. In addition, this study is first to explore the constructed atomistic AChE model of *P*. *americana*, and its possible mode of inhibition by 2,3-dimethylmaleic anhydride through *in-silico* binding analyses. Further, the molecule showed significant impairment in the antioxidant enzymes. Therefore, our study reveals that 2,3-dimethylmaeic anhydride elicits toxicity in *S*. *oryzae* and *P*. *americana* primarily by AChE inhibition along with oxidative imbalance.

## Methods

### Insects

Mixed-age cultures of rice weevil (*Sitophilus oryzae* L.) were collected at ware house of Food Corporation India, Imphal, Manipur, India and reared on whole wheat (*Triticium aestivum*). Whereas American cockroaches (*Periplaneta americana* L.) were collected from Insect Culture centre, at Department of Food Protectant and Infestation Control, CSIR-Central Food Technological Research Institute, Mysore, Karnataka, India and reared in plastic tubes with harborages, containing broken wheat and biscuits, and water was provided ad libitum. The cockroaches were maintained at 23.6 ± 2.5 °C, 70% relative humidity and a photoperiod of 12:12 (Light: Dark). Adults of *S*. *oryzae* (3–5d) were used for the experiments. Experiments were carried out in the laboratory at 27 ± 2 °C and 70 ± 5% r.h.^21,42^.

### Chemicals

*AChE*, acetylthiocholine iodide (ATCI), 5,5-dithio-bis-2-nitrobenzoic acid (DTNB), reduced glutathione (GSH), pyridostigmine bromide, 1-chloro-2,4-dinitrobenzene (CDNB) and Folin’s reagent were procured from Sigma chemical Co. (St. Louis, MO, USA). Trichloroacetic acid (TCA), hydrogen peroxide, sodium di-hydrogen phosphate, sodium hydroxide and sodium carbonate were procured from Sisco Research Laboratory, Mumbai, India. 2,3-dimethylmaleic anhydride is isolated and characterised (purity, 96%) from root stock of *Colocasia esculenta* var esculenta (L.) Schott as reported earlier^21^.

### Insecticidal activity

Knockdown of insects defined as the state of intoxication and partial paralysis with lack of movement which usually precedes death by exposure to 2,3-dimethylmaleic anhydride was done by fumigation using adults of the cockroach and rice weevil. Five adult cockroaches or 30 adults of rice weevil of known age were released into 0.85-l desiccators that served as the fumigation chambers. In each desiccator, a Whatman No. 1 filter circle (9 cm size) was placed to serve as an evaporating surface for injecting the isolated compound. For each species, there were four replicates for each dose of the isolated compound, with equal number of untreated control replicates. The dosages ranged from 0.2 to 20 µg/L, and the effective dosages were chosen based on trial experiments. Four replicates were used for each dosage. KD_50_ (50% knockdown in 24 h exposure) were determined from the dose-response data using probit regression analysis^43^.

### Inhibition of AChE

*In vivo* inhibition of AChE in relation to the toxicity of 2,3-dimethylmaleic anhydride was investigated in cockroaches.

#### Dose-response study

Insects were exposed to KD_25_, KD_50_, and KD_99_ (1.3, 3.0 and 5.9 µg/L for *S*. *oryzae*, 5, 10 and 20 µg/L for *P*. *americana* respectively) doses of 2,3-dimethylmaleic anhydride in the fumigant bioassay^21^. The doses were selected based on the results of toxicity of 2,3-dimethylmaleic anhydride to American cockroach. Respective batches of solvent treated controls were also employed. After 60 min exposure insects were removed. In case of cockroach the neural ganglion were dissected from head out and frozen for enzyme assay, where as in case of stored grain insects whole insect was homogenate and stored at −20 °C for enzyme assay.

#### Time-course study

Insects were exposed to single KD_50_ (10 µg/L for *P*. *americana* and 3.0 µg/L for *S*. *oryzae*) dose of 2,3-dimethylmaleic anhydride in the fumigation bioassay and removed at various exposures time intervals (30, 60, 90, 120 and 150 min). Solvent (ethanol) treated groups served as control. After various time intervals of exposure, insects were removed and the insect tissues were dissected as described above for enzyme assay. Thereafter, homogenate was centrifuged at 10000 rpm for 10–12 min at 4 °C in a table-top refrigerated centrifuge, and the supernatant was separated and used for the further experiments.

### *In vivo* inhibition of Acetylcholinesterase (AChE) in relation to knockdown effect

Inhibition of Acetylcholinesterase (AChE) was estimated by ELISA microplate reader following the modified method of Galgani and Bocquene^44^ which is based on the method of Ellman *et al*.^45^. The reaction was carried out in a 96 vial micro plate and reaction mixture containing the suitable amount supernatant and DTNB in Tris-HCl buffer (100 mM, pH 8.0) was prepared. Thereafter, acetylcholine iodide (ATCI) (10 µl of 0.1 M solution) was added to reaction mixture and change in absorbance was monitored over 3 min in a microplate reader at 405 nm and at 25 °C. The primary AChE inhibition assay was replicated at least four times. The percentage AChE inhibition of the bioactive compound was calculated according to the following formula:

$$\mathrm{Inhibition}\,( \% )=[1-\frac{sample\,reaction\,rate}{blank\,reaction\,rate}]\times 100$$

*In vitro* inhibition of AChE by 2,3-dimethylmaleic anhydride in the neural ganglion of cockroaches, whole insect homogenate of stored grain insects (crude preparation) was studied. The enzyme was pre incubated with 2,3-dimethylmaleic anhydride (0.5 µM–10 µM) along with or without inhibitor (pyridostigmine bromide) at 37 °C for 30 min and the inhibition of AChE was determined.

The type of enzyme inhibition was studied kinetically, in neural ganglion tissue homogenate. The reaction mixture as described above was preincubated with various concentration of with or without inhibitor (2,3-dimethylmaleic anhydride at 5 mM, 10 mM and 15 mM). The residual activity at each of the inhibitor concentration was assayed at various substrate concentrations. The nature of inhibition was determined from the data by double-reciprocal or Linweaver-Burk plot^46^.

## Effect on insect antioxidant enzyme system

### Superoxide dismutase activity (SOD)

Superoxide dismutase (SOD) activity was measured using pyrogallol (2 mM) auto-oxidation as described by Marklund and Marklund^45,47^. The reaction mixture contained pyrogallol in 0.1 M Tris buffer (pH 8.2) and the insect homogenate. The reaction was started by adding the substrate and the absorbance was read at 420 nm for 3 min at an interval of 1 min and results were expressed as unit/mg protein. The amount of the enzyme that inhibits auto-oxidation by 50% is referred as one unit of enzyme activity.

### Reduced glutathione (GSH)

Glutathione (GSH) content was estimated by the method of Ellman^48^, 10% insect tissue homogenates were prepared in 5% w/v TCA, centrifuged at 2000 rpm for 10 min and supernatant (GSH) was mixed with 10 mM DTNB in 0.1 M phosphate buffer (pH 8.0). The mixture was incubated for 10 min at room temperature and the color was read at 412 nm. Glutathione content was calculated by a standard curve and expressed as µg/mg protein.

### Catalase activity (CAT)

Catalase (CAT) activity was assayed by the method of Aebi^49^. The reaction mixture contained 3% H_2_O_2_ in 0.05 M phosphate buffer (pH 7.0). The reaction was started by the addition of 100 μl of enzyme and the change in absorbance was read at 240 nm for 3 min and activity was expressed as n mole H_2_O_2_/min/mg protein.

Protein content was measured by the method of Lowry *et al*.^50^ using BSA as the standard.

### Comparative homology modeling

Acetylcholinesterase (AChE) protein sequence (Accession Number: ALJ10969.1) of *P*. *americana* was retrieved from GenBank in FASTA format, scanned for conserved domains and other putative substrate binding sites using NCBI CDD^51^. SWISS-MODEL (http://swissmodel.expasy.org/), an automated comparative protein modelling server, was used to generate the 3D model by querying the protein sequence as target. The server generated homology models by performing a target-template sequence alignment via searching template structures in Protein Data Bank (PDB) and SWISS-MODEL template library (SMTL) repositories using blastp and HHBlits algorithms^52^. The best structural model was selected based on Global Model Quality Estimation (GMQE) and QMEAN scoring functions, which has been trained specifically for SWISS-MODEL^53^. Stereochemical quality and accuracy of the predicted models were analysed using PROCHECK at PDBsum server (http://www.ebi.ac.uk/thornton-srv/databases/pdbsum/Generate.html), ProQ^54^ and ProSA-web^55,56^.

### Molecular docking

The structural coordinates of 2,3-dimethylmaleic anhydride (PubChem CID: 13010), was downloaded from NCBI PubChem compound database (https://pubchem.ncbi.nlm.nih.gov/) in SDF format and converted to PDB format using Open Babel^57^. The predicted structural model of AChE was analysed for possible ligand binding pockets using CASTp server^58^. Docking simulations were performed using AutoDock 4.2.6^59^ incorporated in MGL Tools version 1.5.6 (The Scripps Research Institute). Rigid docking was used in this study, where protein is treated as a rigid molecule and ligand as flexible. Polar hydrogen atoms were added to the protein and non-polar hydrogen were merged. Gasteiger charge was added to the macromolecule. The grid size was set to 100 Å × 100 Å × 100 Å with a spacing of 0.636 Å to cover entire binding surfaces and pockets of AChE. During the docking, 100 conformations were considered with Lamarckian genetic algorithm search, other assorted parameters were set to the default values of the AutoDock 4.2.6 program. Cluster analyses of these conformations were performed on the basis of their respective root mean square deviation (RMSD) in BIOVIA Discovery Studio visualizer^60^. Top populated conformation clusters from the analysis were selected for comparison with major CASTp pockets and previously designated substrate binding site with catalytic triad of the predicted AChE model. Impending binding cavity was selected based on the top cluster of AutoDock and largest pocket by CASTp lie on top with the substrate binding site. Another set of docking simulations on this resulted binding site was performed by setting up a smaller grid box, 50 Å × 48 Å × 48 Å with a spacing of 0.375 Å and by keeping the rest of the procedures same as earlier. The best docked conformation of 2,3-dimethylmaleic anhydride was selected based on lowest binding energy. Furthermore, CHARMm force-field with smart minimizer (2000 steps) was used to minimize the docked complex, considering Generalized Born with Molecular Volume (GBMV) implicit solvent model. Total and individual interaction energy (IE) possessed by the amino acid residues was calculated. UCSF Chimera^61^ package was used to perform molecular graphics and analyses on the resulted docked complex. Schematic diagrams of the interactions were depicted using LIGPLOT program^62^.

### Statistical analysis

KD_25_, KD_50_ and KD_99_ were determined by Probit analysis^43^. The data were analysed with one-way ANOVA (*p* < 0.05) observed by Newman-Keuls test using Statplus 2007 software and computer program SAS (version 6.12, SAS Institute Inc. Cory, NC, USA).

## Electronic supplementary material

Supplementary Information
