# Supplementary material for: Biochemical efficacy, molecular docking and inhibitory effect of 2, 3-dimethylmaleic anhydride on insect acetylcholinesterase
Source: Sci Rep. 2017 Oct 2;7:12483. doi: 10.1038/s41598-017-12932-0 (PMC5624869; doi:10.1038/s41598-017-12932-0)
Supplement: Supplementary file 1 — Supplementary Information [file 41598_2017_12932_MOESM1_ESM.pdf]

Supplementary Information

Biochemical efficacy, molecular docking and inhibitory effect of 2, 3-dimethylmaleic anhydride on insect acetylcholinesterase

Kabrambam D. Singh[1]. Rajendra K. Labala[2], Thiyam B. Devi[1], Ningthoujam I. Singh[1], Heisnam D. Chanu[1], Sonia Sougrakpam[1], Bunindro S. Nameirakpam[1], Dinabandhu Sahoo[3] and Yallappa Rajashekar[1*]

[1]*Insect Resources Laboratory, Animal Resources Programme, Institute of Bioresources and Sustainable Development, Department of Biotechnology, Govt. of India, Takyelpat, Imphal-795001, Manipur, India*

[2]*Distributed Information Sub-Centre, Institute of Bioresources and Sustainable Development, Department of Biotechnology, Govt. of India, Takyelpat, Imphal-795001, Manipur, India*

[3]*Microbial Resources Programme, Institute of Bioresources and Sustainable Development, Department of Biotechnology, Govt. of India, Takyelpat, Imphal-795001, Manipur, India*

[*]**Corresponding author = Dr. Yallappa Rajashekar [1]***

Scientist-C

Institute of Bioresources and Sustainable Development

Department of Biotechnology, Govt. Of India

Takyelpat Institutional Area, Imphal-795001

Manipur, India.

Email; rajacftri@yahoo.co.in

Telephone: 91 8415902539

Supplementary Figures Page No. 2-3

Supplementary Table Page No. 4

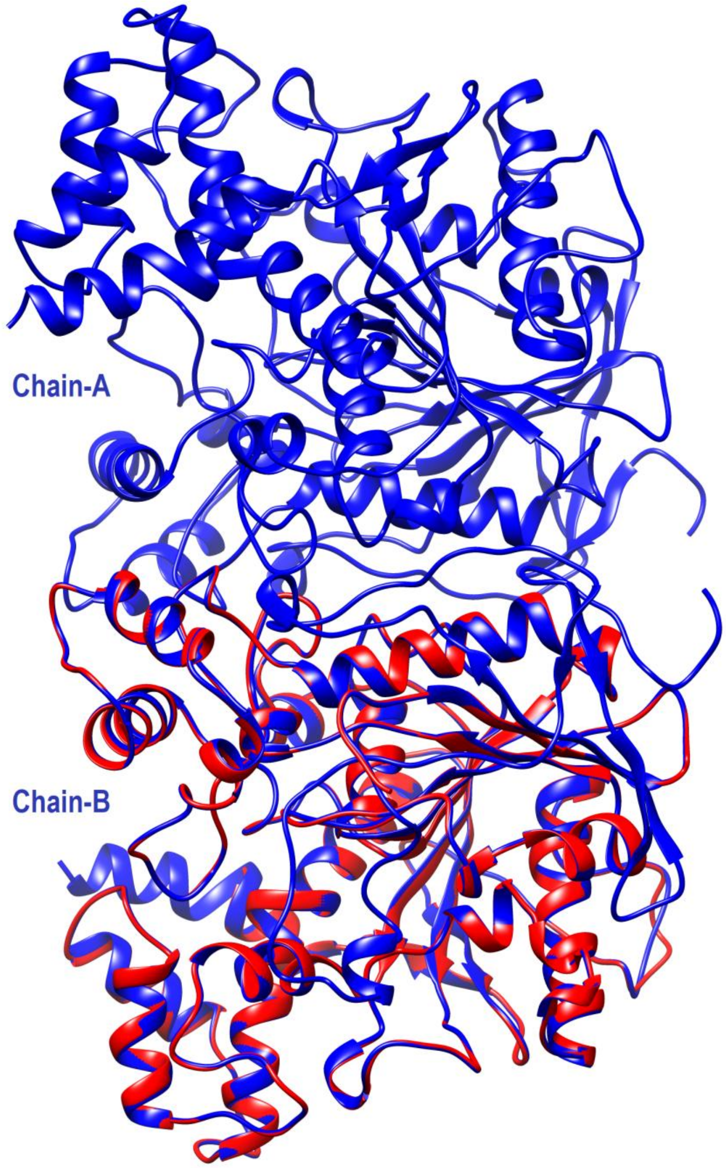

Supplementary Fig. 1| Target AChE model (red) superimposed with the template AChE Catalytic Subunit of *Anopheles gambiae* (PDB ID: 5X61, Chain B) (blue).

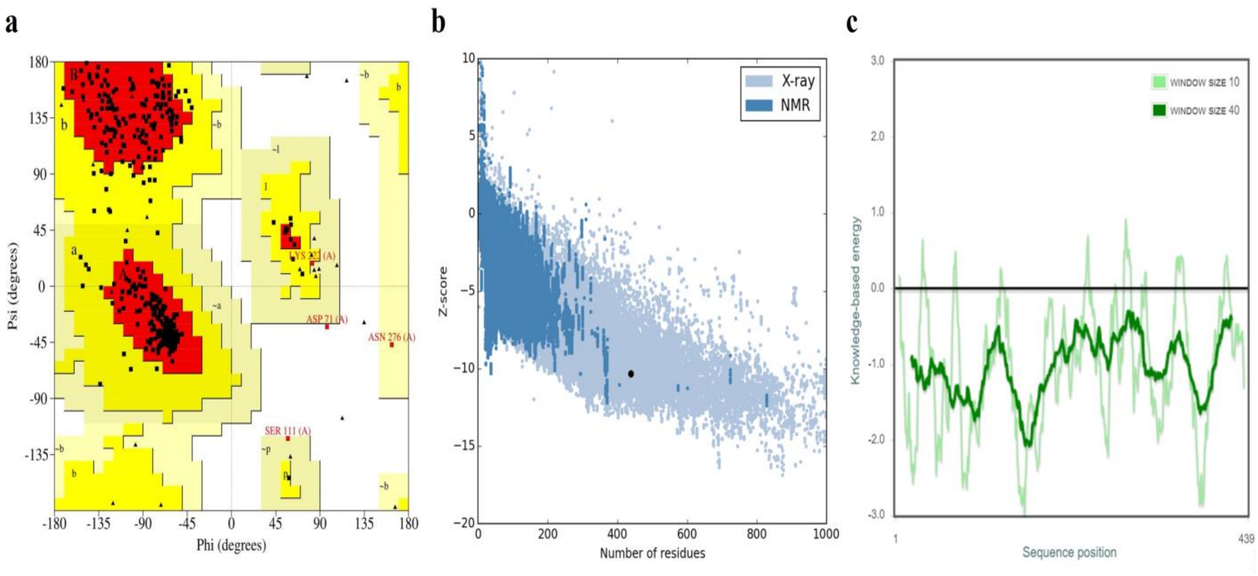

Supplementary Fig. 2| 3D model validation results: a) Ramachandran plot analysis, indicating residues in the favoured regions (red), allowed regions (yellow), generously allowed regions (light yellow) and disallowed regions (white). **b)** Z-score (highlighted as a black dot) is displayed in a plot that contains the Z-scores of all experimentally determined protein chains currently available in PDB. Groups of structures from different sources (X-ray and NMR) are distinguished by different colours (light- and dark-blue, respectively). **c)** Plot of single residue energies, where window sizes of 40 and 10 residues are distinguished by dark- and light-green lines, respectively. Positive values indicate problematic or erroneous parts of the structure.

Supplementary Table. 1| Potential binding sites: Pockets and potential binding sites evidenced by CASTp analysis and AutoDock simulations.

**Pocket Name**	**Area ($Å^2$)**	**Volume ($Å^3$)**	**Amino acid residues involved**
CP1 (by CASTp)	839.2	1140.5	TRP26, PHE28, GLY29, GLY30, GLY31, TYR33, SER34, GLY35, LEU39, VAL41, TYR42, GLU110, SER111, TRP144, TRP192, LEU195, GLY196, ILE197, CYS198, GLU199, PHE200, TYR240, PHE241, TYR244, TYR245, PHE311, PHE334, HIS336, SER338, GLY340, ASN341, PRO342, TRP343, MET350, HIS351, GLY352, ASP353, ILE355, ASN356, TYR357, TYR369
CP2 (by CASTp)	670.4	1108.4	PRO1, LEU2, SER3, GLU4, CYS6, PHE28, TYR33, SER34, GLY35, SER36, GLN59, TYR60, ARG61, VAL62, ALA63, SER64, LEU65, GLY66, PHE67, LEU68, ASP74, VAL75, PRO76, GLY77, ALA79, LEU159, ILE176, LEU179, ARG180, LYS182, ALA184, THR185, LEU187, VAL188
CP3 (by CASTp)	623.7	733.3	PHE334, HIS336, HIS372, GLU375, LEU376, ARG379, MET380, ARG382, TYR383, PRO393, SER394, MET395, GLU397, ASP398, THR400, TRP401, THR402, ALA403, THR404, TYR405, LEU417, THR418, LEU419, ASP420, VAL421, ASN422, SER423, THR424
AC4 (by AutoDock)	NA	NA	ARG155, ARG158, LEU159, ASN190, GLU191, GLY193, LEU195, PRO201, PHE202

*Underlined are substrate binding site residues including catalytic triad
